# B-cell Prolymphocytic Leukemia: Case Report and Challenges on a Diagnostic and Therapeutic Forefront

**DOI:** 10.7759/cureus.5629

**Published:** 2019-09-11

**Authors:** Bikramjit S Bindra, Harpreet Kaur, Shellsea Portillo, Oluwadunni Emiloju, Katherine Garcia de de Jesus

**Affiliations:** 1 Internal Medicine, Government Medical College and Hospital, Chandigarh, IND; 2 Internal Medicine, Albert Einstein Medical Center, Philadelphia, USA; 3 School of Medicine, Catholic University of Honduras, San Pedro Sula, HND; 4 Internal Medicine, St. Barnabas Hospital Health System / Albert Einstein College of Medicine, Bronx, USA

**Keywords:** b-cell prolymphocytic leukemia, prolymphocytic leukemias

## Abstract

B-cell prolymphocytic leukemia (B-PLL) is a rare malignancy of mature B-cells with characteristic morphologic, immunophenotypic, cytogenetic, and molecular features characterized by late onset (median age 69 years), an aggressive clinical course, refractoriness to chemotherapy, and median survival of around three years. Treatment is influenced by the presence or absence of specific high-risk genetic mutations like 17P/TP53 deletion, the presence of which translates into poor prognosis. Patients without 17P deletion, who are <70 years, without significant co-morbidities, are initially treated with a combination chemotherapy regimen used for chronic lymphocytic leukemia (CLL) such as fludarabine, cyclophosphamide, and rituximab. On the other hand, patients with a 17P deletion, age >70 years, with multiple co-morbidities, receive ibrutinib or alemtuzumab as the initial therapy. Relapsed or refractory cases are managed with BCL-2 signaling inhibitors like venetoclax. We discuss the case of an 84-year-old male with B-PLL (positive TP53 mutation), resistant to ibrutinib therapy, with extremely high white blood cell (WBC) counts, thus creating a dilemma regarding the best treatment in the second-line setting.

## Introduction

Prolymphocytic leukemias (PLLs) are rare mature lymphoid disorders of B- and T-cells exhibiting characteristic features and an aggressive clinical course [1]. Relevant cytogenetic abnormalities in cases of B-cell prolymphocytic leukemia (B-PLL) include MYC rearrangements and overexpression, deletions of 17p/TP53 mutations, and deletions of 13q14. Among these, 17p deletion or TP53 mutations are associated with a worse prognosis due to primary resistance to first-line chemotherapy drugs [2]. Due to a scarcity of data, chronic lymphocytic leukemia (CLL) guidelines are used to guide appropriate treatment regimens in cases of B-PLL. Despite advances in the understanding of the biology and pathogenesis, the prognosis remains poor, with early relapses and short overall survival time [3].

## Case presentation

An 84-year-old African American male presented with progressively increasing fatigue, weakness, and a 10 lbs unintentional weight loss over the past five months. His past medical history was significant for multiple co-morbid conditions, including hypertension, hyperlipidemia, end-stage renal disease (on peritoneal dialysis since the past 1.5 years), chronic obstructive pulmonary disease, and depression. On physical examination, he appeared visibly fatigued. Repeat blood work was significant for a white blood cell (WBC) count of 96.3x10^3^/μL, red blood cell (RBC) count of 3.24 x10^6^/μL, hemoglobin 10 gm/dl, hematocrit 31.5%, red cell distribution width 15.4%, lactate dehydrogenase 537 IU/L, folate 4.0 ng/ml, and decreased kidney function. Peripheral blood smear showed >60% prolymphocytes. A contrast-enhanced computed tomography (CT) scan of chest/abdomen and pelvis showed marked splenomegaly with an ill-defined area of decreased enhancement, concerning for a malignancy. This necessitated a bone marrow biopsy, which revealed numerous prolymphocytes (74%). Morphologically, the cells were large, almost double the size of normal lymphocytes, with a prominent central round nucleus, condensed nuclear chromatin, and a scarce, faintly basophilic cytoplasm. There were no nuclear indentations, cytoplasmic hairy projections, or villi. The Ki-67 proliferation index was >40%, pointing towards a diagnosis of PLL. Flow cytometry was positive for CD45, CD19, CD20, CD22, CD23, kappa light chain, HLA-DR, and CD5 and negative for CD10, CD38, CD34, lambda light chain, and other T-cell myeloid markers, consistent with a B-cell lymphoproliferative disorder. Fluorescence in situ hybridization was positive for 17p(TP53) and 13q14 deletions. Based on the histopathology, immunohistochemistry, and genetic analysis, a diagnosis of B-PLL was made.

**Figure 1:**
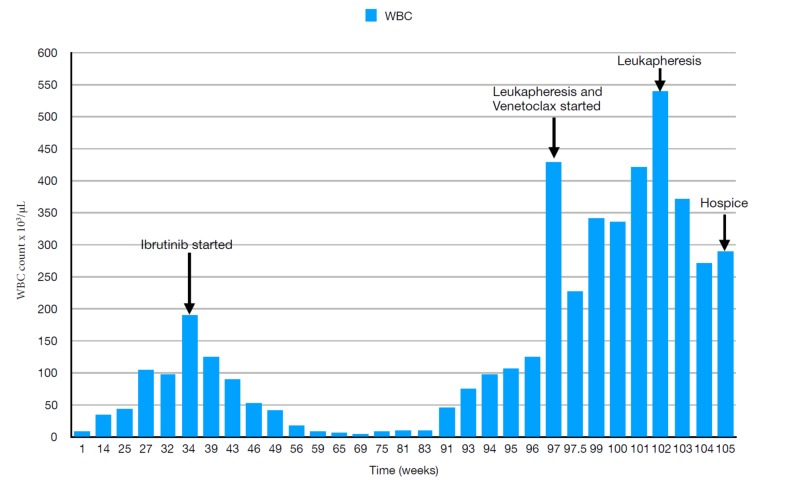
Variation in the WBC count during the course of therapy Abbreviations: White blood cell (WBC)

The treatment was extrapolated from the CLL guidelines, and the patient was started on first-line therapy with Ibrutinib 420 mg/day. The patient had an initial dramatic response to ibrutinib, with WBCs decreasing from 189 x10^3^/μL to less than 10x10^3^/μL over the next three months and attaining a stable value after that for almost a year (Figure 1 shows the variation in WBC count during the course of therapy). One year after starting ibrutinib, routine blood work revealed WBC count >50x10^3^/μL, with a peripheral smear showing excess prolymphocytes. Flow cytometry findings were consistent with a relapse of B- PLL. Again, CLL guidelines were used for guiding further management, and venetoclax was added to the regimen based on a five-week gradual ramp-up schedule from 20 mg/day to 400 mg/day. During the initial ramp-up, the patient's lymphocytosis worsened, with WBCs reaching up to 430 x10^3^/μL. Due to concerns for cerebrovascular and cardiopulmonary complications from profound lymphocytosis, the patient underwent urgent inpatient leukapheresis. His dose was ramped up in subsequent weeks to 400 mg/day. In the meantime, the patient underwent several leukapheresis sessions on an outpatient basis to keep the WBC count in check. Subsequently, the patient was started on rituximab, 100 mg/m^2^. He developed a life-threatening anaphylactic reaction while on rituximab, precluding its further use. He was salvaged using fluids and vasopressors and was admitted. During this time, the patient spontaneously developed altered sensorium. Blood work during the course of admission consistently revealed a WBC count >350x10^3^/μL. Blood counts and kidney function were closely monitored throughout the treatment. Considering the poor response to therapy and a worse prognosis, the patient was referred to hospice care.

## Discussion

B-PLL is an extremely rare lymphoid malignancy, comprising less than 1% of B-cell leukemias [1]. The disease course is characterized by refractoriness to chemotherapy, with a median survival of around three years [4]. The 'prolymphocytes' are actually mature, activated B cells, which invade the peripheral blood, bone marrow, and spleen. Earlier, B-PLL was considered a variant of CLL but, following the World Health Organization (WHO) classification in 2008 and the revised WHO classification of lymphoid neoplasms in 2016, B-PLL was recognized as a distinct mature B-cell entity [4]. As per the French-American-British group, the number of prolymphocytes circulating in the peripheral blood must exceed 55% of the total circulating cells. However, in reality, this number is often greater than 90% [5-6]. Our patient was initially thought to have CLL with increased prolymphocytes (CLL/PLL, defined as between 15% and 55% prolymphocytes). But the peripheral blood smear had more than 60% prolymphocytes, and bone marrow aspirate showed 74% prolymphocytes, thus fulfilling the diagnostic criterion of PLL.

Patients typically present with a rapidly rising white blood cell count of >100 x10^3^/μL, massive splenomegaly, and rapidly declining cell lines, leading to anemia and thrombocytopenia [7]. Systemic B-symptoms like fevers, night sweats, and weight loss are also common at the time of presentation. Peripheral lymphadenopathy is not a common finding (only seen in 1/3rd of the patients) and, if present, is often small in volume [4]. In the setting of a relapse, B-PLL can have some atypical presentations, including central nervous system involvement, refractory hypercalcemia, and even an intramuscular mass as reported by Aude et al. [3].

The diagnosis of B-PLL is often challenging because of the considerable overlap with other mature B-cell leukemias and lymphomas. Morphologically, B-prolymphocytes have a large size (approximately twice the size of small lymphocytes), moderately condensed nuclear chromatin, a prominent central nucleolus, and a characteristic round or oval nucleus. Cytoplasmic hairy projections or villi are absent, thus differentiating it from hairy cell leukemia variant (HCL) and splenic marginal zone lymphoma (SMZL). Even though a careful examination of peripheral blood morphology can aid in the diagnosis of B-PLL, it is insufficient, requiring immunophenotypic and genetic analysis of the peripheral blood for a more concrete diagnosis. On immunophenotyping, B-PLL, being a tumor of monoclonal B-cells, expresses bright surface IgM +/- IgD, bright surface Ig kappa or lambda light chain, bright CD20, and CD19, CD22, CD79a, and FMC7. This can also help in differentiating B-PLL from CLL, which generally has a dim expression of surface Ig and CD20 [3-5]. Also, there is an absence of expression of CD11c, CD103, CD10, CD25, and cyclin D1, which further helps in distinguishing B-PLL from other chronic lymphoid neoplasms with a leukemic presentation such as T-cell prolymphocytic leukemia, mantle cell lymphoma (MCL), follicular lymphoma, lymphoplasmacytic lymphoma, and HCL [7].

B-PLL, as such, does not have a distinct cytogenetic signature, and only a few cytogenetic studies have been reported in the literature. The most frequently reported cytogenetic abnormalities include MYC rearrangements and overexpression (seen in approximately 50% of cases) [3], deletions of 17p (the chromosomal arm that carries the TP53 gene), and TP53 mutations with a reported incidence of 53% as per Lens et al. [2], and, finally, the deletion of 13q14, which occurs in about 25% of cases.

B-PLL generally has a poor prognosis (median survival of three years). Similar to CLL, the presence of the TP53 mutation is considered a poor prognostic factor and is associated with resistance to chemotherapy and short overall survival times [3-4]. Lymphocyte count >100 x10^3^/μL and anemia < 11 g/dl at the time of diagnosis have also been seen to be associated with shorter survival [8]. Our patient was positive for 17p(TP53) and 13q14 deletions, had a high lymphocyte count, and anemia at the time of presentation, thus hinting towards a guarded prognosis.

There is a scarcity of data to guide therapy in B-PLL cases. Therefore, due to the lack of clear-cut treatment guidelines, the regimens used for CLL are often employed for treating B-PLL. Understandably, responses in such cases are often partial and rarely durable. Once again, extrapolating from the CLL guidelines, deletion of the 17p or TP53 mutation is considered a high-risk genetic feature and is often used to guide therapy. Patients without a 17p deletion or TP53 mutations are initially treated with a combination of fludarabine, cyclophosphamide, and rituximab. Conversely, patients with a 17p deletion or TP53 mutations have primary resistance to purine analog/alkylator-based therapy, thus making the aforementioned chemotherapeutic agents less effective [3]. Newer drugs like alemtuzumab, which is an anti-CD52 monoclonal antibody, and Ibrutinib, which is an inhibitor of Bruton tyrosine kinase, have shown positive results in recent case reports [9]. Our patient had an excellent initial response to ibrutinib therapy with WBC counts decreasing to less than 10x10^3^/µL over one month of starting therapy. The patient was able to maintain a stable count of less than 15,000/µL over the next 10 months. Allogeneic hematopoietic cell transplantation is also an option but is limited to young patients, has a good performance status, and shows a good response to initial therapy [10]. Given the history of multiple comorbid conditions and the advanced age of presentation, our patient was not an ideal candidate for bone marrow transplantation.

Relapsed and refractory cases of B-PLL are particularly challenging and there is an extreme paucity of literature, which is limited to case reports and series. Nonetheless, serial therapies, similar to those used for CLL, are employed for such scenarios. There are encouraging case reports available in which clinical remission was attained using inhibitors of BCR signaling (ibrutinib, idelalisib) and BCL2 signaling (venetoclax) [11-12]. Again, we would like to emphasize that such incidences of successful treatment of B-PLL are numbered and most of the data is available in the form of discrete case reports/series and is, at best, an extrapolation from trials in CLL.

## Conclusions

B-PLL usually presents with a rapidly rising WBC count of >100x10^3^/μL, massive splenomegaly, anemia, and thrombocytopenia. Similarities with other mature B-cell malignancies like MCL, CLL, HCL, and SMZL can often complicate the diagnosis. However, distinctions based on histology, immunophenotyping, and genetic analysis are often helpful. Treatment options are numbered, and most of them are borrowed from the CLL regimens. Given the rarity of B-PLL, there is a paucity of data evaluating these treatment options and much of the treatment regimens is either based on case reports/series or extrapolated from trials in CLL. Dedicated trials are needed for developing treatment options for this less common, aggressive disease entity.
